# Supplementary material for: Non-Reproducibility of Oral Rotenone as a Model for Parkinson’s Disease in Mice
Source: Int J Mol Sci. 2022 Oct 21;23(20):12658. doi: 10.3390/ijms232012658 (PMC9604506; doi:10.3390/ijms232012658)
Supplement: Supplementary file 1 [file ijms-23-12658-s001.zip › ijms-1963985-supplementary.pptx]

## Slide 1
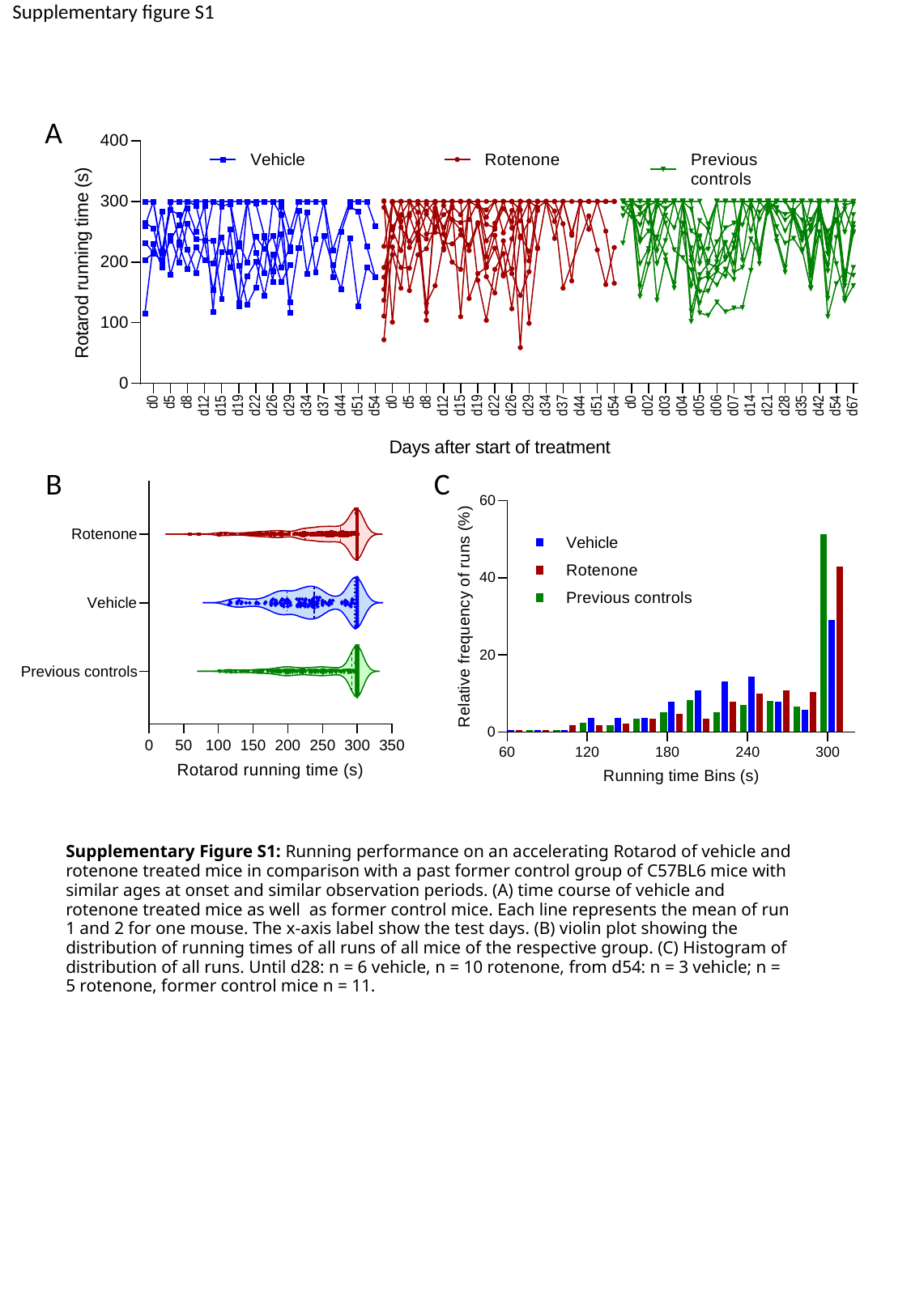

Supplementary figure S1
A
B
C
Supplementary Figure S1: Running performance on an accelerating Rotarod of vehicle and rotenone treated mice in comparison with a past former control group of C57BL6 mice with similar ages at onset and similar observation periods. (A) time course of vehicle and rotenone treated mice as well as former control mice. Each line represents the mean of run 1 and 2 for one mouse. The x-axis label show the test days. (B) violin plot showing the distribution of running times of all runs of all mice of the respective group. (C) Histogram of distribution of all runs. Until d28: n = 6 vehicle, n = 10 rotenone, from d54: n = 3 vehicle; n = 5 rotenone, former control mice n = 11.

## Slide 2
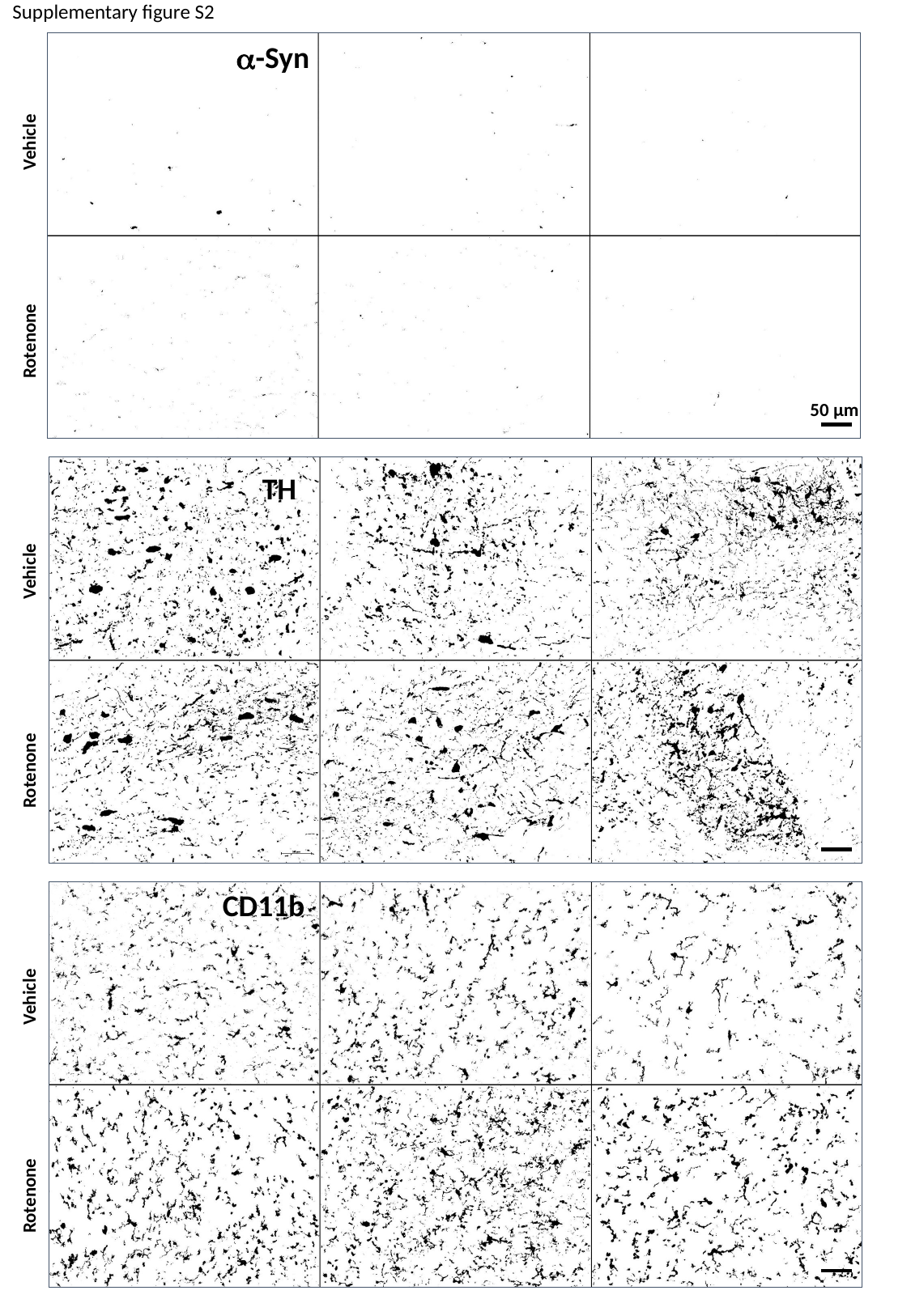

Supplementary figure S2
a-Syn
Vehicle
Rotenone
50 µm
TH
Vehicle
Rotenone
CD11b
Vehicle
Rotenone

## Slide 3
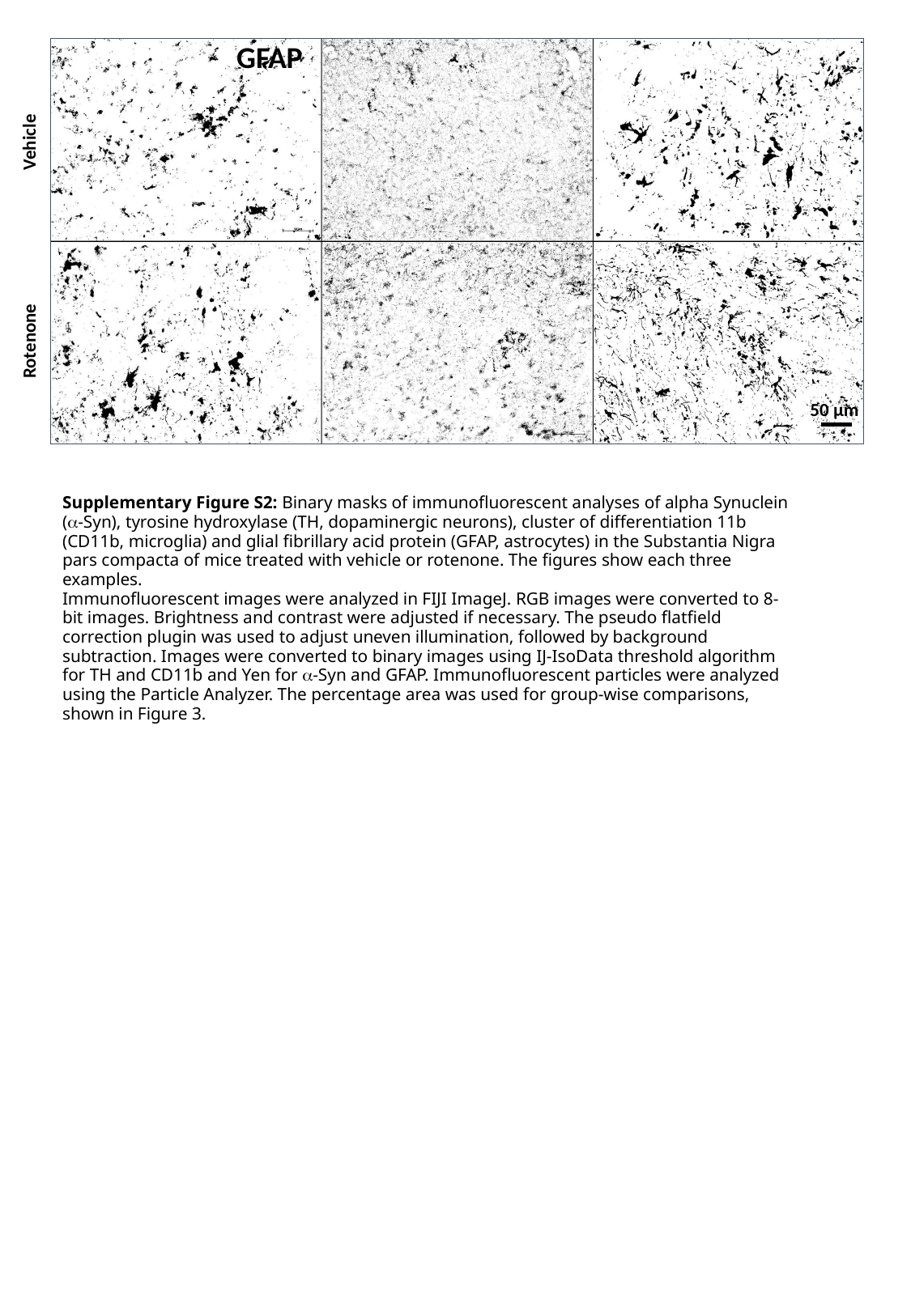

GFAP
Vehicle
Rotenone
50 µm
Supplementary Figure S2: Binary masks of immunofluorescent analyses of alpha Synuclein (a-Syn), tyrosine hydroxylase (TH, dopaminergic neurons), cluster of differentiation 11b (CD11b, microglia) and glial fibrillary acid protein (GFAP, astrocytes) in the Substantia Nigra pars compacta of mice treated with vehicle or rotenone. The figures show each three examples.
Immunofluorescent images were analyzed in FIJI ImageJ. RGB images were converted to 8-bit images. Brightness and contrast were adjusted if necessary. The pseudo flatfield correction plugin was used to adjust uneven illumination, followed by background subtraction. Images were converted to binary images using IJ-IsoData threshold algorithm for TH and CD11b and Yen for a-Syn and GFAP. Immunofluorescent particles were analyzed using the Particle Analyzer. The percentage area was used for group-wise comparisons, shown in Figure 3.
